# A Large Community-Acquired Lung Abscess Caused by Pseudomonas aeruginosa: Diagnostic and Therapeutic Challenges

**DOI:** 10.7759/cureus.105657

**Published:** 2026-03-22

**Authors:** Bassem Al Hariri, Roaa Thaher, Zobaida Osman, Yasin Zada, Anas Sheikh Al Ard Khanji

**Affiliations:** 1 College of Medicine, Qatar University, Doha, QAT; 2 College of Medicine, Weill Cornell Medicine - Qatar, Doha, QAT; 3 Internal Medicine, Hamad Medical Corporation, Doha, QAT; 4 Internal Medicine, Hamad General Hospital, Hamad Medical Corporation, Doha, QAT

**Keywords:** antimicrobial therapy, bronchoscopy, community-acquired infections, lung abscess, multidisciplinary care, pseudomonas aeruginosa

## Abstract

In immunocompetent hosts without healthcare exposure, *Pseudomonas aeruginosa* is an uncommon pathogen causing community-acquired lung abscesses. These infections are usually polymicrobial and include oropharyngeal flora. We describe a case of a 52-year-old man who had previously been in good health but presented with a four-day history of anorexia, malaise, and fever. Fever (38.0°C), tachycardia (107 bpm), and decreased breath sounds with dullness to percussion over the left lower lung region were noted on physical examination. Significant leukocytosis (11.3 × 10³/μL) and elevated C-reactive protein (252.8 mg/L) were found on laboratory investigations. A sizable (9.3 × 9.6 cm) cavitary lesion with peripheral enhancement in the left lower lobe was confirmed by chest radiography and contrast-enhanced computed tomography, consistent with a primary lung abscess. *Pseudomonas aeruginosa* was identified as the causative pathogen by bronchoscopy and bronchoalveolar lavage, whereas tuberculosis was ruled out by polymerase chain reaction and negative smears. Clinical improvement occurred within 72 hours of initiating targeted intravenous piperacillin-tazobactam 4.5 g every six hours after infectious disease consultation. Without the need for percutaneous drainage, the patient completed a six-week course of antibiotics, including a 14-day intravenous course followed by four weeks of oral ciprofloxacin 750 mg twice daily. This case emphasizes three key lessons: (1) the value of multidisciplinary management in balancing prolonged medical therapy and interventional drainage for large abscesses; (2) the critical role of bronchoscopic sampling in identifying atypical pathogens and guiding targeted antimicrobial therapy; and (3) the importance of maintaining diagnostic suspicion for lung abscess in patients with constitutional symptoms even in the absence of classic respiratory complaints.

## Introduction

A lung abscess is a necrotic cavitary lesion of the pulmonary parenchyma caused by a microbial infection. Although the widespread use of antibiotics has reduced its incidence, with current estimates suggesting that it accounts for a small fraction of pulmonary infections, diagnostic and therapeutic difficulties still exist [1]. Classic risk factors include periodontal disease, alcoholism, neurological disorders affecting swallowing, and immunocompromised states. Polymicrobial infections involving anaerobic and aerobic bacteria originating from the oropharyngeal flora are commonly responsible for community-acquired lung abscesses, and standard management typically involves prolonged antibiotic therapy targeting these organisms [2]. Despite being a well-known pathogen in infections linked to healthcare settings and in patients with structural lung disease or immunocompromised hosts, *Pseudomonas aeruginosa* remains a rare cause of community-acquired lung abscess in immunocompetent individuals [3]. To highlight the diagnostic process, microbiological analysis, and multidisciplinary therapeutic approach that resulted in a satisfactory outcome without the need for invasive drainage, we present a case of a large primary lung abscess caused by *P. aeruginosa* in an otherwise healthy adult.

## Case presentation

A 52-year-old Indian male manual laborer with no noteworthy medical history arrived at the ER after experiencing worsening constitutional symptoms for four days. His main complaints were generalized myalgia, acute anorexia, and fever. Interestingly, he did not report dyspnea, hemoptysis, or cough. He denied IV drug use, alcohol consumption, recent travel, or sick contacts. Otherwise, the review of systems was unremarkable. He lived in shared housing with other employees.

At the time of initial evaluation, the patient was conscious, alert, and appeared non-toxic. Vital signs included a blood pressure of 138/84 mmHg, respiratory rate of 18 breaths per minute, oxygen saturation of 97% on room air, temperature of 38.0°C, and heart rate of 107 beats per minute. His BMI was 29.4 kg/m². Cardiopulmonary auscultation over the left mid-to-lower lung fields revealed a systolic murmur and, more importantly, a marked reduction in breath sounds with dullness to percussion. The right lung was clear. The rest of the physical examination was unremarkable.

Significant leukocytosis (11.3 × 10³/μL) with neutrophilic predominance (78.6%) and significantly elevated CRP (252.8 mg/L) were found on the initial laboratory tests (Table 1). The basic metabolic panel revealed normal renal function and mild hyponatremia (134 mmol/L). Procalcitonin was elevated at 0.40 ng/mL. HbA1c was 5.8%, making undiagnosed diabetes less likely as a risk factor, and HIV antigen/antibody testing was non-reactive.

**Table 1:** Key laboratory parameters on admission and during follow-up. This table shows the evolution of the patient’s key laboratory values from admission to day 10 of hospitalization. The marked decrease in inflammatory markers (CRP) and WBC count correlates with the clinical improvement observed after initiation of targeted antibiotic therapy.

Parameter	Reference Range	Admission (Day 1)	Follow-up (Day ~10)	Clinical Implication
Hemoglobin (g/dL)	13.0-17.0	12.5	10.3	Mild anemia on admission, progressing to chronic disease.
WBC Count (×10³/µL)	4.0-11.0	11.3	8.2	Initial leukocytosis indicating acute infection, trending down with therapy.
Neutrophils (%)	40-75	78.6	77.3	Predominant neutrophilia, consistent with acute bacterial infection.
CRP (mg/L)	<5.0	252.8	59.7	Markedly elevated, confirming significant inflammatory burden; decreasing with treatment.
Sodium (mmol/L)	135-145	134	132	Mild hyponatremia, likely due to SIADH from pulmonary infection.
Creatinine (µmol/L)	64-104	92	81	Normal renal function throughout.
Procalcitonin (ng/mL)	<0.05	0.4	-	Moderately elevated, supporting bacterial etiology.
HIV Ag/Ab Combo	Non-reactive	Non-reactive	-	Ruled out immunocompromising conditions.
HbA1c (%)	<5.7	5.8	-	Ruled out undiagnosed diabetes as a predisposing factor.

**Figure 1:**
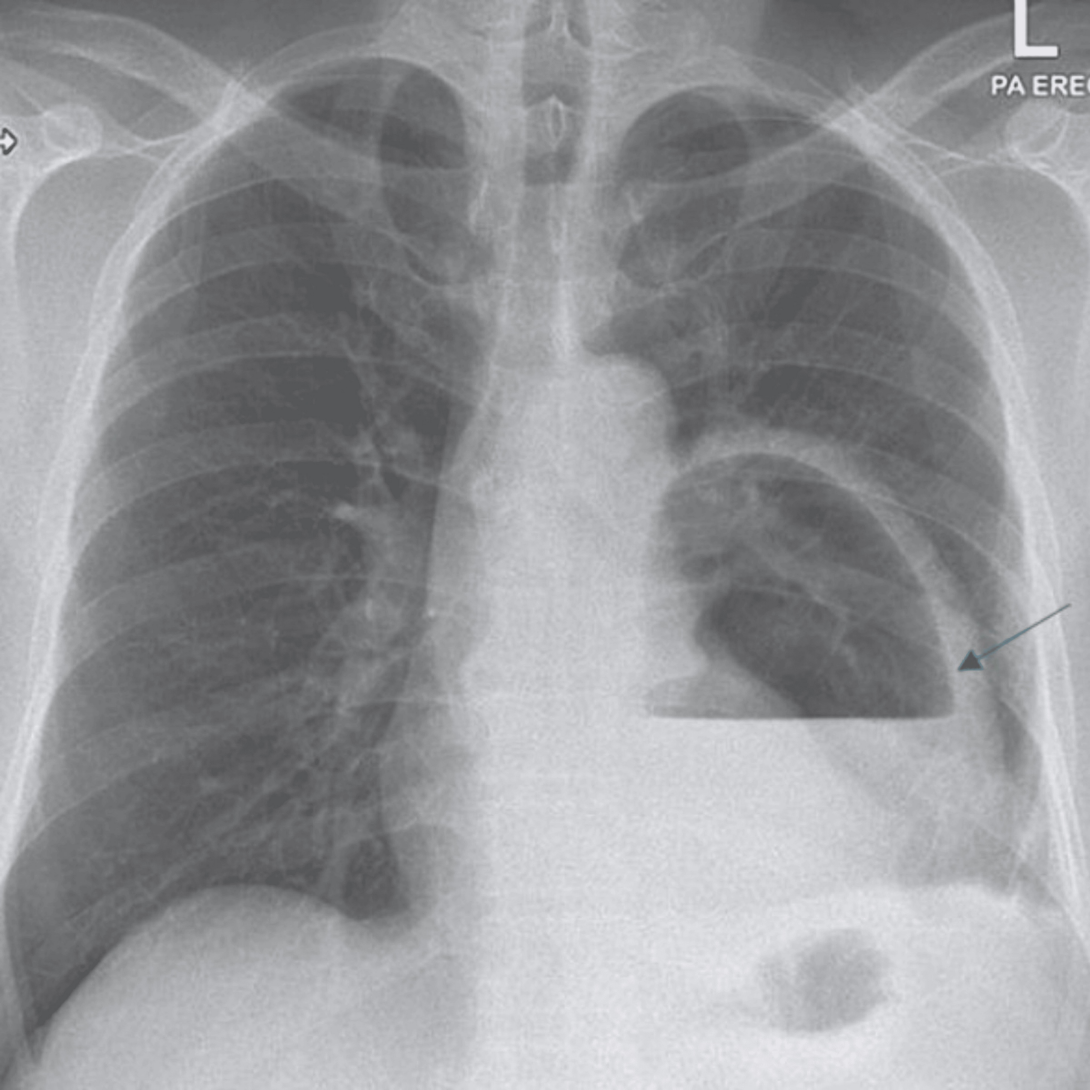
Chest X-ray. Posteroanterior chest radiograph demonstrating a large cavitary lesion in the left lower lung zone. A prominent air-fluid level (arrow) is visible within the cavity, a characteristic finding of a lung abscess.

A posteroanterior chest radiograph demonstrated a large cavitary lesion with an air-fluid level in the left lower lung zone (Figure 1).

**Figure 2:**
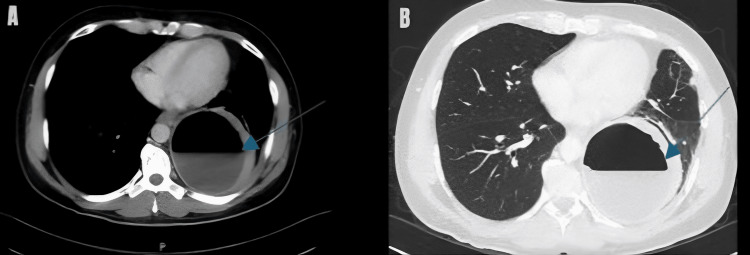
Contrast-enhanced CT thorax. Axial (A) and coronal (B) contrast-enhanced CT images of the chest definitively characterize the left lower lobe abscess. The images demonstrate a large (9.3 × 9.6 cm), thick-walled cavitary lesion with peripheral enhancement (arrows), confirming the diagnosis of a primary lung abscess and excluding pleural involvement.

Contrast-enhanced CT of the thorax definitively characterized a 9.3 × 9.6 cm, thick-walled, peripherally enhancing cavitary mass in the posterior segment of the left lower lobe, consistent with a primary lung abscess (Figures 2A-2B). The study incidentally noted multiple calcified gallstones without acute pathology.

Due to the extent of the abscess and the need for a conclusive microbiological diagnosis, specifically to rule out tuberculosis, flexible bronchoscopy with bronchoalveolar lavage (BAL) was performed on the fifth day of the hospital stay. Bronchoscopy showed inflammatory changes and circumferential narrowing of the left lower lobe bronchus. BAL fluid analysis revealed a negative Mycobacterium tuberculosis polymerase chain reaction result and negative acid-fast bacilli smears. Bacterial culture of the BAL fluid grew *P. aeruginosa* susceptible to piperacillin-tazobactam and ciprofloxacin. Blood cultures remained sterile. A transthoracic echocardiogram showed normal cardiac function and no valvular vegetations.

Antibiotic therapy and clinical course

Management followed a multidisciplinary approach involving pulmonology, infectious disease, and interventional radiology services. Upon admission, the patient was started on empirical IV antibiotics: ceftriaxone 2 g every 24 hours and metronidazole 500 mg every 8 hours.

On hospital day four, following infectious disease consultation and based on the clinical suspicion of a Gram-negative infection (prior to definitive culture results), antibiotic therapy was modified to IV piperacillin-tazobactam 4.5 g (4 g piperacillin / 0.5 g tazobactam) every 6 hours. This empiric adjustment was later confirmed to be appropriate when the BAL culture grew piperacillin-tazobactam-susceptible *P. aeruginosa*.

The treatment consensus established a six-week total antibiotic course, consisting of an intensive IV phase with piperacillin-tazobactam 4.5 g every 6 hours for 14 days, completed during hospitalization, followed by an oral step-down phase with ciprofloxacin 750 mg twice daily for the remaining 4 weeks, completed as an outpatient.

Within 72 hours of starting piperacillin-tazobactam, the patient's fever subsided, demonstrating a clear clinical and biochemical response. Consistent improvement was seen in inflammatory markers; by the tenth day of hospitalization, CRP had dropped to 59.7 mg/L from 252.8 mg/L at admission. Because of this favorable clinical trajectory, image-guided percutaneous drainage was deemed unnecessary. The patient was discharged from the hospital on day 14, with close outpatient infectious disease follow-up, to complete the oral ciprofloxacin course.

## Discussion

In the context of managing a primary lung abscess, this case offers a number of clinically noteworthy elements that merit careful consideration. In contrast to the classic symptoms traditionally associated with lung abscess, such as foul-smelling sputum or a definite preceding aspiration event, the patient's presentation was noticeably subtle [1]. This pattern is consistent with changes in epidemiology in the modern era, where classic presentations are less distinct. The physical examination findings of unilateral dullness and absent breath sounds provided the diagnostic clue that prompted rapid radiographic evaluation. This emphasizes the importance of maintaining a high level of diagnostic suspicion when a patient presents with persistent fever and constitutional symptoms, even in the absence of obvious respiratory complaints.

The radiological findings illustrate the current diagnostic imaging paradigm. Contrast-enhanced CT proved definitive, providing crucial information that guided management decisions, including the abscess size (>6 cm), the presence of an air-fluid level, the thick enhancing wall indicating active infection, and the absence of pleural involvement or empyema, even though chest radiography had provided the first clue [4]. These CT features successfully differentiated the abscess from other cavitary diseases, including necrotizing pneumonia, localized empyema, and cavitating malignancy. Such accurate anatomical characterization is essential for both prognostic evaluation and the planning of possible interventional procedures.

Most importantly, the microbiological etiology was both surprising and educational. A rare cause of community-acquired lung abscess, *P. aeruginosa* is typically linked to immunocompromised states, bronchiectasis, or healthcare exposure [3]. Its isolation in this patient, who had no such risk factors, has important implications. First, it draws attention to a limitation of empirical antibiotic regimens that typically target anaerobes and oral streptococci. Second, it strongly supports aggressive diagnostic sampling to obtain culture material directly from the abscess cavity. By avoiding oropharyngeal contamination and providing a reliable specimen for culture, bronchoscopy with BAL, as carried out in this instance, was beneficial [5]. This strategy directly enabled targeted therapy and improved outcomes. The negative tuberculosis workup further supported the bacterial etiology and was particularly important given the epidemiological background and radiographic appearance.

A review of the literature reveals only rare reports of community-acquired *P. aeruginosa* lung abscesses in immunocompetent hosts. Similar to our case, these reports consistently describe large abscess cavities, the need for bronchoscopic or transthoracic sampling for definitive diagnosis, and successful outcomes with prolonged, targeted anti-pseudomonal therapy. This comparison contextualizes our findings within the limited existing evidence and reinforces the favorable prognosis achievable with medical management alone in such cases.

The management approach is a prime example of contemporary therapeutic principles. The initial broad-spectrum empirical antibiotics, ceftriaxone and metronidazole, targeting common oropharyngeal microorganisms, were an appropriate early strategy. Treatment was logically escalated to an anti-pseudomonal beta-lactam, piperacillin-tazobactam 4.5 g every 6 hours, after clinical concern for treatment failure and the subsequent detection of *P. aeruginosa*. The choice of piperacillin-tazobactam 4.5 g every 6 hours is worth noting. This dosing regimen achieves the time above the minimum inhibitory concentration (MIC) required for bacterial eradication in deep-seated parenchymal infections, offering optimal pharmacokinetic/pharmacodynamic coverage against *P. aeruginosa*.

Susceptibility testing supported the switch to oral ciprofloxacin 750 mg twice daily, which is a typical step-down strategy that takes advantage of the drug's high oral bioavailability (~70-80%) and superior lung tissue penetration. To prevent recurrence, the prolonged treatment course, four weeks of oral therapy after 14 days of IV therapy, for a total of six weeks, aligns with current guidance for complicated parenchymal infections, particularly for abscesses larger than 6 cm, to ensure complete resolution and reduce the risk of recurrence [6]. Evidence supporting prolonged therapy for large abscesses (>6 cm) to ensure full resolution and reduce the risk of recurrence is consistent with this duration.

Attention should be paid to the decision to forgo image-guided percutaneous drainage. Although drainage is usually advised for abscesses larger than 6 cm or those that do not improve with medical therapy [6], our patient showed early clinical and biochemical improvement within 72 hours of receiving appropriate anti-pseudomonal therapy. This experience reaffirms that size alone does not require invasive intervention; rather, a trial of targeted medical therapy is reasonable in stable patients. The multidisciplinary framework involving pulmonology, infectious disease, and interventional radiology created a safety net that allowed for timely escalation should it become necessary.

It was rightly concluded that the incidental gallstones were not contributory. Monitoring during convalescence is warranted because the mild persistent anemia (hemoglobin 10.3 g/dL) seen during follow-up is suggestive of anemia of chronic inflammation, a common consequence of serious infection.

Due to the nature of single-case observations, this case report has limitations. The therapeutic strategy should be tailored to the specific circumstances of each patient and to local patterns of antibiotic resistance, as these results may not be generalizable to all patients with lung abscesses.

## Conclusions

This case describes an immunocompetent adult with a massive community-acquired lung abscess caused by *P. aeruginosa*, an atypical pathogen in this population. Three key messages emerge for clinical practice: (1) lung abscess should remain in the differential diagnosis for patients presenting with fever and constitutional symptoms, even in the absence of classic respiratory complaints or risk factors; (2) cross-sectional imaging is essential for diagnostic confirmation and characterization; and (3) obtaining reliable microbiological specimens, ideally via bronchoscopy, is critical for guiding targeted, long-term antimicrobial therapy and optimizing outcomes. The multidisciplinary approach integrating pulmonology, infectious disease, and interventional radiology expertise proved instrumental in achieving successful medical management with a six-week antibiotic regimen (14 days of intravenous piperacillin-tazobactam 4.5 g every 6 hours, followed by 4 weeks of oral ciprofloxacin 750 mg twice daily) without requiring invasive drainage.
